# Screening for HIV, hepatitis B and syphilis on dried blood spots: A promising method to better reach hidden high-risk populations with self-collected sampling

**DOI:** 10.1371/journal.pone.0186722

**Published:** 2017-10-20

**Authors:** Inge H. M. van Loo, Nicole H. T. M. Dukers-Muijrers, Rosalie Heuts, Marianne A. B. van der Sande, Christian J. P. A. Hoebe

**Affiliations:** 1 Department of Medical Microbiology, Maastricht University Medical Centre, Maastricht, The Netherlands; 2 Care and Public Health Research Institute (CAPHRI), Maastricht University, Maastricht, The Netherlands; 3 Department for Sexual Health, Infectious Diseases and Environmental Health, Public Health Service South Limburg, Geleen, The Netherlands; 4 Centre Infectious Disease Control, National Institute for Public Health and the Environment, Bilthoven, The Netherlands; 5 Julius Center, University Medical Center Utrecht, Utrecht, The Netherlands; 6 Institute of Tropical Medicine, Antwerp, Belgium; Centers for Disease Control and Prevention, UNITED STATES

## Abstract

**Introduction:**

Many people at high risk for sexually transmitted infections (STIs), e.g., men who have sex with men (MSM), are not optimally reached by current sexual health care systems with testing. To facilitate testing by home-based sampling or sampling in outreach setting we evaluated dried blood spots (DBS), a method for self-collected blood sampling for serological screening of HIV, hepatitis B (HBV) and syphilis. The aims of this study were to assess the acceptability and feasibility of self-collected DBS and to compare the test results for screening of HIV, HBV and syphilis from DBS with blood drawn by venous puncture.

**Methods:**

DBS were collected from men who have sex with men (MSM), visiting the STI clinic of the public health service South Limburg (n = 183) and HIV positive and HBV positive patients (n = 34), visiting the outpatient clinics of the Maastricht University Medical Centre in the period January 2012–April 2015. The 93 first participating MSM visiting the STI clinic were asked to fill in a questionnaire about the feasibility and acceptability about self-collection of DBS in a setting without going to a health care facility and were asked to collect the DBS themselves. Serological screening tests for HIV (HIV combi PT, Roche), HBV (HBsAg, Roche) and syphilis (*Treponema pallidum Ig*, Biokit 3.0) were performed on DBS and on blood drawn by venous puncture, which was routinely taken for screening.

**Results:**

In total 217 participants were included in the study with a median age of 40 years (range between 17–80). Of MSM 84% agreed that it was clear and easy to do the finger-prick, while 53% agreed that it was clear and easy to apply the blood onto the DBS card. Also, 80% of MSM would use the bloodspot test again. In 91% (198) of DBS, sufficient material was collected to perform the three tests. No difference was observed in DBS quality between self-collected DBS and health care worker collected DBS. For HIV (n = 195 DBS-serum pairs) sensitivity and specificity were 100%. For HBV the sensitivity for HBsAg (n = 202) was 90% and specificity was 99%. For syphilis (n = 191) the sensitivity of the DBS was 93% with a specificity of 99%. Analysis of the DBS of HIV positive participants (n = 38) did show similar test performance for HBV and syphilis as in HIV negatives.

**Conclusion:**

DBS is an acceptable self-sampling method for MSM, as there was no difference in DBS quality in self-collected and health care worker collected DBS. Test performance, i.e., its high sensitivity (>90%) and specificity (>99%) measures show that DBS is a valid alternative for venous blood puncture. Especially when DBS is combined with home-collected sampling for *Chlamydia trachomatis* and *Neisseria gonorrhoeae*, complete STI screening can be done in outreach setting and/or home-collected sampling in MSM.

## Introduction

For the control of sexually transmitted infections (STI) timely testing and adequate treatment strategies are essential to prevent adverse health outcomes and reduce or halt transmission. Still many infected people remain untested and untreated [1–3]. This may be because they do not attend care or because they are not adequately tested when in care. For example, while STI clinic guidelines recommend regular testing in men who have sex with men (MSM), the reality is that MSM are infrequently tested, only partially tested or not tested at all [4–6]. Even, HIV positive MSM may remain hidden to care, because they are not visiting regular (STI) care services and/or STI care is often considered beyond the focus of the HIV practitioner [6, 7]. Further, MSM who contact their GP may not be completely tested, such as would have been the case when they were tested according to STI clinic guidelines (i.e. on human immunodeficiency virus (HIV), syphilis, Hepatitis B virus (HBV), and anorectal and urogenital and/or oropharyngeal *Chlamydia trachomatis* (CT) and *Neisseria gonorrhoeae* (NG) [8].

To increase STI testing beyond regular STI care, many target group specific interventions have been set up to increase testing behavior in key populations like MSM. These include the use of web based outreach strategies combined with home-collection of screening samples for testing [9–11]. It has been shown that self-sampling can be a feasible and effective alternative for key populations that are currently not visiting STI clinics or accessing other regular health care. Self-sampling especially introduced in STI screening programs can be a valuable addition to current STI control [10, 12–16].

Till now self-based testing interventions were mainly focused on CT and NG testing in heterosexual individuals as these samples have been shown to be as easy, acceptable and valid compared to samples taken by a health care provider [12, 13]. However, for screening of HIV, HBV and syphilis, a care provider still needs to draw a venous blood sample hampering home-sampling. Self-taken blood sampling procedures are hardly available in routine STI care settings in industrialized countries. An alternative self-sampling blood test could positively impact efficiency of STI control in MSM. Also in outreach screening and in hard to reach populations in partner testing or enhanced screening of potentially affected contacts during outbreaks for e.g. syphilis self-sampling may be of added value.

A method for self-collected sampling of blood samples, more commonly used in developing countries, is sampling by dried blood spots (DBS) with a finger prick. Blood droplets are absorbed on a filter paper, which subsequently can be sent to a laboratory by regular mail [17–19].

Previous studies showed that DBS results for HIV, HBV and syphilis are as reliable as venous blood puncture [17–24]. Most studies are, however, performed in settings different from ours, e.g. hospital setting instead of self-collected sampling, different climatic circumstances, so that transport conditions are different.

In this study we assessed the acceptability and feasibility of self-collection of DBS in MSM visiting an STI clinic and we evaluated the performance of DBS for serological screening of HIV, HBV and syphilis compared to blood drawn by venipuncture in our regular routine setting of our STI clinic and HIV and hepatitis outpatient clinic of our hospital. A valid and acceptable alternative for serum could likely have great impact to reach hidden MSM with testing as an addition to regular care.

## Methods

### Study populations

DBS were collected from MSM, visiting the STI clinic of the public health service South Limburg (PHS) and among HIV and/or HBV infected patients, visiting the outpatient clinics for HIV and/or hepatitis of the Maastricht University Medical Centre (MUMC) in the period January 2012—April 2015. In total 217 individuals participated; 183 visited the STI clinic and 34 the outpatient clinics. The participants of the outpatient clinics were included in the study to purposefully increase the number of HIV and HBV positive DBS samples.

### Questionnaire about feasibility and acceptability

The first 93 (51%) MSM who participated in de DBS study, visiting the STI clinic were asked to fill in a questionnaire about the feasibility and acceptability about self-collection of DBS. These individuals were asked to collect the DBS themselves to imitate the sampling situation at home as much as possible. They were provided an instruction scheme with several steps depicted by icons and a clear picture of the DBS card with the 5 circles (cross section 1.5 cm) for sampling. Three completely filled spots were needed to perform 3 tests. The DBS from the other participants were taken by health-care providers to compare self-sampling with sampling by a health care provider. The questionnaire included questions whether they (completely) agreed with statements (5-items) on the experience of self-collection, the instructions for use, the future use of DBS, comparing different care settings and social acceptance.

### Routine diagnostics for HIV, Treponema pallidum and HBV

HIV Ag/Ab, *Treponema* Ig and HBsAg are essential tests to screen for active infections and used for this purpose in current care. Although the national policy is vaccinating all MSM for HBV not all MSM are fully vaccinated yet and therefore HBsAg screening is included in this study.

Screening for HIV infection is done with a fourth generation screening HIV Ag/Ab test (HIV combi PT, Roche, Basel, Switzerland). The result of the HIV Ag/Ab test gave an index value, which was interpreted as follows: < 0.9 is negative, > = 0.9 and <1.1 is grey zone and > = 1.1 is positive. Grey zone and positive screening tests were confirmed by an immunoblot (MP diagnostics, Santa Anna, California, USA).

The algorithm for syphilis screening on serum was as follows: For seronegative individuals screening was performed with an anti-Treponemal antibody (*T*. *pallidum* Ig) test (*Treponema pallidum*, Biokit 3.0, Barcelona, Spain). Borderline or positive *T*. *pallidum* Ig tests were confirmed by TPPA (Fujirebio Diagnostic inc. Malvern, PA, USA) and FTA-abs (Trepo-spot IF, Biomerieux, Lyon, France) for confirmation of the syphilis infection and RPR (RPR reditest, Biokit, Barcelona, Spain) to determine stage and activity of infection. Discrepant combination of results were sent to a reference laboratory (National Institute for Public Health and the Environment) for confirmation with immunoblot. The result of the *T*. *pallidum* Ig test was indicated as an index value, which was interpreted as follows: <0.9 was negative, > = 0.9 and <1.1was grey zone and > = 1.1 was positive.

In routine setting screening for HBV on serum was done with testing HBsAg for active HBV infection (HBsAg II, Roche, Basel, Switzerland). In case of a positive HBsAg test, anti HBc, anti-HBs, HBeAg, and anti-HBe (anti-HBc, anti-HBs II, HBeAg, anti-HBe, Roche, Basel, Switzerland) were additionally determined in serum. If necessary a HBsAg confirmation test was used (Biomerieux, Lyon, France). The result of the HBsAg gave an index value, which was interpreted as follows: For HBsAg < 0.9 was negative, > = 0.9 and <1.1 was grey zone and > = 1.1 was positive.

The DBS results were compared with the results from routine diagnostic test algorithms. This means that (low) positive screening tests in serum that could not be confirmed in the confirmation assays were interpreted as negative. Thus the conclusive result based on both regular screening test and confirmatory test (gold standard) were used to compare with DBS, which were tested with the screening tests only (i.e. HIV Ag/Ab, *Treponema* Ig and HBsAg). For confirmatory tests following positive screening in DBS a secondary sample (i.e. venous blood) would be required.

### Elution from DBS

DBS were drawn from the 3^rd^ or 4^th^ finger by puncture with a lancet. For this study a card with 5 spots with a diameter of 15 mm should be filled with blood. After taking the DBS the cards were air-dried for at least 10 minutes. The cards were transported to the laboratory within 48 hrs. in sealed bags.

The cards were kept at 4°C until analysis. Analysis was done within 5 days. Eight mm discs were punched and placed into a microtube. Discs for HIV Ag/Ab and HBsAg were eluted in PBS– 0.5% tween buffer and for *T*. *pallidum* Ig discs were eluted in diluent of the assay (Biokit 3.0, Barcelona, Spain). Two discs were placed in 250 μl buffer and incubated for 1 hour on a rotation table. After centrifuging the eluate was directly used for the tests.

### Medical ethical approval

The medical ethics committee of the Maastricht University Medical Centre (Maastricht, the Netherlands) approved the study (12-4-040). All participants were asked prior to voluntary participation to give their written informed consent.

### Statistical analysis

Descriptive statistics were performed using IBM® SPSS Statistics (V.23.0.0, IBM, Somers, New York, USA). Chi-square test was used for comparing self-collected and health care provider collected DBS and coefficient of determination (R^2^) were used for comparing analytical indexes of the test results of DBS and sera.

## Results

### Study participants

The median age of the participants (n = 217) was 40 years with a range between 17–80 years. The majority of the participants (202/217, 93%) were of male sex. From the STI clinic 183 MSM participated and from the out-patient clinic 19 men and 15 women.

The DBS contained variable amounts of blood per spot and variable numbers of blood spots were filled (up to five). In 198 (91.2%) of participants 3 spots were sufficiently filled to perform 3 tests. From 5 and 1 participants, respectively 2 and 1 test(s) could be performed due to low amount of blood. Thirteen DBS cards could not be used for testing because the blood volume on the DBS was insufficient for testing. There was no statistically significant difference in self-sampling (n = 88) or sampling by a health care provider (n = 129) in the number of tests that could be performed (Chi^2^ P = 0.73).

### Feasibility and acceptability

Ninety three MSM participants filled in the questionnaire on feasibility and acceptability (Table 1). Notable was that the majority of men agreed that it was clear (84%) and easy (86%) to do the finger-prick, while lower percentages agreed that it was clear and easy to apply the blood onto the card. Also, the majority of men would use the bloodspot test again, but only half mentioned that sending DBS over the postal mail would be fine. Few of these men, who came for a clinic visit and then were confronted with a DBS, stated that they would prefer DBS over clinic visit. Slightly less than half of the men thought that their friends would use DBS and might also prefer home-based sampling over a clinic visit.

**Table 1:** Acceptance of DBS testing and attitudes towards future use in 93 MSM undergoing DBS test and attending an STI clinic for regular STI care.

Instructions and experience of use	(completely) agree *
It is clear how to do the finger-prick	84.2% (n = 80)
It is easy to do the finger-prick	86.3% (n = 82)
How to apply the blood on the card is clear	69.5% (n = 66)
To apply the blood on the card is easy	53.7 (n = 51)
It is unpleasant to do the finger-prick	26.3% (n = 25)
**Future use**	
A bloodspot test is a good initiative	57.9% (n = 55)
I would do the bloodspot test again	80.0% (n = 76)
To send my bloodspot over the postal mail is fine with me	50.5% (48)
A test result by email or text message is acceptable	89.5% (85)
**Comparing care-settings**	
I prefer a finger-prick over blood drawing by a clinic nurse	8.4% (n = 8)
I prefer to do an STI test at home than to come to the STI clinic	15.8% (n = 15)
A personal talk with a professional nurse is more important to me than able to do a test at home	81.1% (n = 77)
**Social acceptance**	
I think that my friends find an STI test with a bloodspot by the Internet a good initiative	49.5% (n = 47)
I think that my friends would use the blood spot test at their home	41.1% (n = 39)
I think my friends would prefer to do an STI test at home than to come to the STI clinic	47.4% (n = 45)

Acceptance of DBS testing and attitudes towards future use in 93 MSM undergoing DBS test and attending an STI clinic for regular STI care.

*score 1 and 2 on likert scale 1–5 (completely agree to completely disagree)

### Comparison of DBS and routine diagnostics

For validity of DBS analysis we tested 195 DBS-serum pairs for HIV Ag/Ab, 191 for *T*. *pallidum* Ig and 202 DBS-serum pairs for HBsAg. HIV Ag/Ab showed a 100% sensitivity and specificity (Table 2). For *T*. *pallidum* Ig the sensitivity was 90% with specificity of 99%. The sensitivity for HBsAg was respectively 90% with specificity of 99%.

**Table 2:** Test results of DBS-serum pairs of the validation study.

Test	No. of samples tested	No. samples positive in DBS and serum	No. samples negative in DBS and serum	Sensi-tivity	95%CI; upper and lower limit of sensitivity	Speci-ficity	95% CI; upper and lower limit of specificity
**HIV Ag/Ab**	195	38	157	100%	81%-100%	100%	97%-100%
***T*. *pallidum* Ig**	191	26	160	90%	72%-97%	99%	95%-100%
**HBsAg**	202	17	183	90%	71%-100%	99%	97%-100%

Number of DBS-serum pairs tested and test results of the validation study (95%CI–95% confidence interval).

### Discrepancy analysis

In total 7 (1.2%) out of 588 DBS-serum pairs tested showed a different result in DBS and serum. No discrepant results were observed for HIV Ag/Ab. For *T*. *pallidum* Ig 3 infections were false negative in DBS and 2 borderline false positive in DBS. For HBsAg, 2 discrepant results were found; one false positive in DBS and one false negative in DBS. (Table 3).

**Table 3:** Analysis of discrepant results in DBS and serum.

Test	No. of samples tested	No. of discrepant results	Discrepancy in	Conclusion
***T*. *pallidum* Ig**	191	3	DBS − / serum +	false negative in DBS: confirmation of lues infection with positive TPPA and FTA-Abs
		2	DBS + / serum −	borderline false positive in DBS; serum was negative
**HBsAg**	202	1	DBS − / serum +	HBsAg = 2.2 in serum: chronic HBV carrier in reconvalescence phase
		1	DBS + / serum −	false positive HBsAg = 1.17 index in DBS

Analysis of discrepant results in DBS and serum.

### Index values in DBS and serum

**Fig 1:**
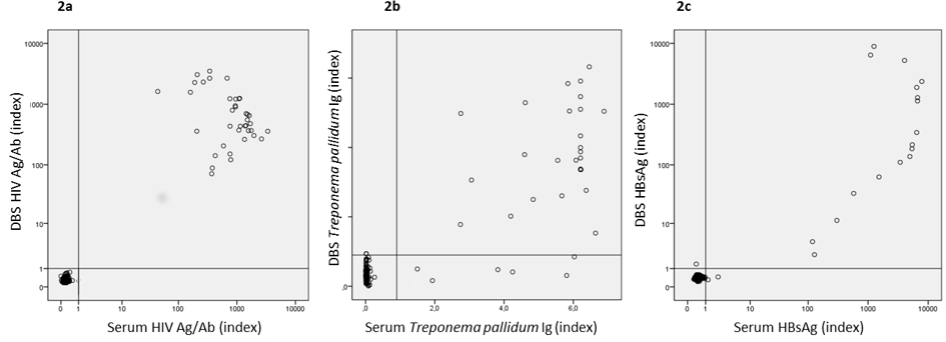
Scatterplots of the index values measured in DBS and serum. Scatterplots of the index values measured in DBS and serum. 2a. shows the log transformed results for HIV Ag/Ab, 2b. the results for *T*. *pallidum* Ig 2c. the log transformed results for HBsAg.

Index values for HIV Ag/Ab, *T*. *pallidum* Ig and HBsAg were compared between DBS and serum (Fig 1). For HIV Ag/Ab and HBsAg a clear distinction between positive and negative results was shown (R^2^ were respectively, 0.92 and 0.90 for HIV Ag/Ab and HBsAg). For *T*. *pallidum* Ig this distinction was less clear, since the number of false negatives in DBS were higher, compared to the HIV and HBsAg index values (see previous paragraph) (R^2^ was 0.75 for *T*. *pallidum* Ig).

### HIV positive participants

In total 38 HIV positive participants were included. Test performance appeared similar in HIV positives with sensitivities of 92% and 100% respectively for *T*. *pallidum* Ig and HBsAg and specificities of 100% for the both tests, although confidence intervals were wide (Table 4).

**Table 4:** Subgroup analysis of DBS-serum pairs in HIV positive participants.

Test	No. of samples tested	No. of samples positive in DBS and serum	No. of samples negative in DBS and serum	Sensi-tivity	95% CI; upper and lower limit of sensitivity	Speci-ficity	95% CI; upper and lower limit of specificity
***T*. *pallidum* Ig**	36	12	24	92%	74–100%	100%	79%–100%
**HBsAg**	38	1	37	100%	3%–100%	100%	91%–100%

Subgroup analysis of DBS-serum pairs in HIV positive participants (95% CI–95% confidence interval).

## Discussion

This study shows that DBS as method for sampling is a valid alternative for venous blood puncture with high sensitivity (>90%) and specificity (>99%) for HIV Ag/Ab, *Treponema pallidum* Ig and HBsAg. DBS is also found acceptable in MSM in our study. To a large extent DBS proved to be likely feasible in routine use since overall 91% of the DBS was adequately taken to perform the three screening tests. Also, there was no difference in the quality of DBS taken by self-sampling compared to DBS taken by a health care provider This makes self-collected sampling with DBS (in combination with additional genital and anorectal CT and NG testing) a feasible approach to reach part of the hidden MSM. It could therefore be a valuable addition to regular care and also could be used for outreach settings [16, 25].

This study is not without limitations. In 70% of the participants who filled in the questionnaire the method was clear and they would do DBS again. Nevertheless, acceptability was measured in a selection of MSM (possibly overestimating acceptance) who attended the STI clinic (possibly underestimation of acceptance as they already came to a clinic and already had received care by a professional). Although we included patients from the HIV and hepatitis out-patient clinics to artificially increase the number of positives analyses, the numbers were still rather small (between 17 and 38), possibly limiting the precision in the sensitivities estimated. Other studies also studied cohorts with various numbers of positives (between 10 to 204) [19, 20, 22, 24, 26]. Further, we have small numbers for subgroup analysis, such as analyses by HIV status.

The quality of DBS may be subject to interobserver bias because the DBS were visually screened before preparation of the eluate. However, we expect this to be a small bias and therefore of minor influence on our results.

HIV Ag/Ab in DBS showed a sensitivity and specificity of 100%. Comparison of the index values in DBS and serum show a good correlation. Similar results were found in other studies showing sensitivities of 100% and specificities of 99.5%-100% in DBS despite the fact that different elution protocols and screening assays were used in these studies [19, 20, 22, 23].

*T*. *pallidum* Ig in DBS in this study showed a sensitivity of 90% with a specificity of 99%. Smit et al. found a sensitivity (94%) with EIA syphilis screening [26]. They found, however, a low specificity of 50%, which may be due to increased non-specific binding in the assay, because they used a longer incubation time.

HBsAg showed acceptable sensitivity of 90% and specificity of 99%. Other studies showed sensitivities of 96–98% and specificities of 100% [17, 20, 22, 24]. Our study showed a slightly lower sensitivity because one low positive HBsAg (index of 2.2) was missed in DBS. This serum was from a chronic HBV carrier in the period of seroconversion from positive to negative. These and other data indicate that the lower detection limit is increased in DBS and low positive levels of HBsAg levels in serum may be missed in DBS [22]. However, this should be no problem in routine screening for active HBV infection (either acute or chronic HBV), because it has been shown that HBsAg levels in chronically HBV infected persons are usually above 100 IU/ml, which can be detected by DBS [27–29]. This is in line with the results of our other HBsAg positive participants (Fig 1).

Several factors should be taken into account to implement DBS as method for sampling. Successful implementation would for example be dependent on whether users understand how to apply the blood samples on the paper and how willing they would be to send the DBS by postal mail. The actual application in care however still needs to be assessed, such as in our ongoing intervention study called PacMan. Although there is room for improving the instructions for taking DBS, the results in this study with respect to the acceptability and feasibility was overall adequate. In 91% of the DBS 3 tests could be performed. One study with a response rate of 90% showed that for HIV screening with DBS 99% of the DBS were adequately taken by self-collection [30]. Yet, in our study 9% were inadequately taken DBS and instructions can be optimized to improve this further.

To be able to implement DBS in routine setting it is also important that storage and transport conditions do not influence the test results. In routine use it will take up to 3 days before DBS will arrive at the laboratory when DBS are home-collected and sent to the laboratory by regular mail. For HIV Ag/Ab and HBsAg storage conditions have been assessed for stability at room temperature up to 200 days [18, 19, 24]. DBS were stable at room temperature for at least one week until 4 weeks [18, 19, 24]. For implementation in routine setting the storage at room temperature does not exceed one week, if DBS are directly sent to the laboratory. Thus, it is not expected that sending DBS by regular mail would harm the test results and it is the most convenient way of transportation of DBS to the laboratory.

In conclusion, DBS as method for self-sampling at home is a valid and likely acceptable alternative for venous blood puncture for STI screening in MSM. It was also feasible to iuse in addition to in routine STI clinic care. The next step is to implement DBS as method for home-collected sampling in combination with self-collection of swabs and/or urine for *CT* and *NG* screening in order to better reach the hidden MSM with STI testing and treatment.

## References

[1] Roth AM, Rosenberger JG, Reece M, Van Der Pol B. Expanding sexually transmitted infection screening among women and men engaging in transactional sex: the feasibility of field-based self-collection. Int J STD AIDS. 2013;24(4):323–8. doi: 10.1177/0956462412472791 ; PubMed Central PMCID: PMCPMC3970701. 23970665 10.1177/0956462412472791 PMC3970701

[2] van Liere GA, Dukers-Muijrers NH, van Bergen JE, Gotz HM, Stals F, Hoebe CJ. The added value of chlamydia screening between 2008–2010 in reaching young people in addition to chlamydia testing in regular care; an observational study. BMC Infect Dis. 2014;14:612 doi: 10.1186/s12879-014-0612-2 ; PubMed Central PMCID: PMCPMC4239384. 25403312 10.1186/s12879-014-0612-2 PMC4239384

[3] Verhaegh-Haasnoot A, Dukers-Muijrers NH, Hoebe CJ. High burden of STI and HIV in male sex workers working as internet escorts for men in an observational study: a hidden key population compared with female sex workers and other men who have sex with men. BMC Infect Dis. 2015;15:291 doi: 10.1186/s12879-015-1045-2 ; PubMed Central PMCID: PMCPMC4517560. 26220287 10.1186/s12879-015-1045-2 PMC4517560

[4] den Heijer CD, van Liere GA, Hoebe CJ, van Bergen JE, Cals JW, Stals FS, et al Who tests whom? A comprehensive overview of Chlamydia trachomatis test practices in a Dutch region among different STI care providers for urogenital, anorectal and oropharyngeal sites in young people: a cross-sectional study. Sex Transm Infect. 2016;92(3):211–7. doi: 10.1136/sextrans-2015-052065 . 26265066 10.1136/sextrans-2015-052065

[5] den Heijer CDJ, Hoebe C, van Liere G, van Bergen J, Cals JWL, Stals FS, et al A comprehensive overview of urogenital, anorectal and oropharyngeal Neisseria gonorrhoeae testing and diagnoses among different STI care providers: a cross-sectional study. BMC Infect Dis. 2017;17(1):290 doi: 10.1186/s12879-017-2402-0 ; PubMed Central PMCID: PMCPMC5397759. 28427377 10.1186/s12879-017-2402-0 PMC5397759

[6] den Daas C, Doppen M, Schmidt AJ, Op de Coul E. Determinants of never having tested for HIV among MSM in the Netherlands. BMJ Open. 2016;6(1):e009480 doi: 10.1136/bmjopen-2015-009480 ; PubMed Central PMCID: PMCPMC4716195. 26758261 10.1136/bmjopen-2015-009480 PMC4716195

[7] Dukers-Muijrers NH, Somers C, Hoebe CJ, Lowe SH, Niekamp AM, Oude Lashof A, et al Improving sexual health for HIV patients by providing a combination of integrated public health and hospital care services; a one-group pre- and post test intervention comparison. BMC Public Health. 2012;12:1118 doi: 10.1186/1471-2458-12-1118 ; PubMed Central PMCID: PMCPMC3537529. 23270463 10.1186/1471-2458-12-1118 PMC3537529

[8] Verlee EL, van Bergen JE, Dekker JH, Boeke AJ, Burgers JS, Bouma M. [Summary of the NHG guideline 'The STD consultation']. Ned Tijdschr Geneeskd. 2014;158:A7277 . 24690519

[9] Theunissen KA, Hoebe CJ, Crutzen R, Kara-Zaitri C, de Vries NK, van Bergen JE, et al Using intervention mapping for the development of a targeted secure web-based outreach strategy named SafeFriend, for Chlamydia trachomatis testing in young people at risk. BMC Public Health. 2013;13:996 doi: 10.1186/1471-2458-13-996 ; PubMed Central PMCID: PMCPMC4015304. 24148656 10.1186/1471-2458-13-996 PMC4015304

[10] Dukers-Muijrers NH, Theunissen KA, Wolffs PT, Kok G, Hoebe CJ. Acceptance of Home-Based Chlamydia Genital and Anorectal Testing Using Short Message Service (SMS) in Previously Tested Young People and Their Social and Sexual Networks. PLoS One. 2015;10(7):e0133575 doi: 10.1371/journal.pone.0133575 ; PubMed Central PMCID: PMCPMC4539363. 26230085 10.1371/journal.pone.0133575 PMC4539363

[11] van den Broek IV, van Bergen JE, Brouwers EE, Fennema JS, Gotz HM, Hoebe CJ, et al Effectiveness of yearly, register based screening for chlamydia in the Netherlands: controlled trial with randomised stepped wedge implementation. BMJ. 2012;345:e4316 doi: 10.1136/bmj.e4316 ; PubMed Central PMCID: PMCPMC3390168. 22767614 10.1136/bmj.e4316 PMC3390168

[12] Hoebe CJ, Rademaker CW, Brouwers EE, ter Waarbeek HL, van Bergen JE. Acceptability of self-taken vaginal swabs and first-catch urine samples for the diagnosis of urogenital Chlamydia trachomatis and Neisseria gonorrhoeae with an amplified DNA assay in young women attending a public health sexually transmitted disease clinic. Sex Transm Dis. 2006;33(8):491–5. doi: 10.1097/01.olq.0000204619.87066.28 . 16547452 10.1097/01.olq.0000204619.87066.28

[13] van der Helm JJ, Hoebe CJ, van Rooijen MS, Brouwers EE, Fennema HS, Thiesbrummel HF, et al High performance and acceptability of self-collected rectal swabs for diagnosis of Chlamydia trachomatis and Neisseria gonorrhoeae in men who have sex with men and women. Sex Transm Dis. 2009;36(8):493–7. doi: 10.1097/OLQ.0b013e3181a44b8c . 19617869 10.1097/OLQ.0b013e3181a44b8c

[14] Fajardo-Bernal L, Aponte-Gonzalez J, Vigil P, Angel-Muller E, Rincon C, Gaitan HG, et al Home-based versus clinic-based specimen collection in the management of Chlamydia trachomatis and Neisseria gonorrhoeae infections. Cochrane Database Syst Rev. 2015;(9):CD011317 doi: 10.1002/14651858.CD011317.pub2 . 26418128 10.1002/14651858.CD011317.pub2 PMC8666088

[15] Odesanmi TY, Wasti SP, Odesanmi OS, Adegbola O, Oguntuase OO, Mahmood S. Comparative effectiveness and acceptability of home-based and clinic-based sampling methods for sexually transmissible infections screening in females aged 14–50 years: a systematic review and meta-analysis. Sex Health. 2013;10(6):559–69. doi: 10.1071/SH13029 . 24160747 10.1071/SH13029

[16] Paudyal P, Llewellyn C, Lau J, Mahmud M, Smith H. Obtaining self-samples to diagnose curable sexually transmitted infections: a systematic review of patients' experiences. PLoS One. 2015;10(4):e0124310 doi: 10.1371/journal.pone.0124310 ; PubMed Central PMCID: PMCPMC4409059. 25909508 10.1371/journal.pone.0124310 PMC4409059

[17] Halfon P, Penaranda G, Mohamed S, Camus C, Khiri H. Nationwide large survey on hepatitis B surface antigen quantification use in real-life clinical practice. Eur J Gastroenterol Hepatol. 2015;27(5):557–60. doi: 10.1097/MEG.0000000000000326 . 25822864 10.1097/MEG.0000000000000326

[18] McAllister G, Shepherd S, Templeton K, Aitken C, Gunson R. Long term stability of HBsAg, anti-HBc and anti-HCV in dried blood spot samples and eluates. J Clin Virol. 2015;71:10–7. doi: 10.1016/j.jcv.2015.07.303 . 26370308 10.1016/j.jcv.2015.07.303

[19] Castro AC, Borges LG, Souza Rda S, Grudzinski M, D'Azevedo PA. Evaluation of the human immunodeficiency virus type 1 and 2 antibodies detection in dried whole blood spots (DBS) samples. Rev Inst Med Trop Sao Paulo. 2008;50(3):151–6. . 18604415 10.1590/s0036-46652008000300004

[20] Ross RS, Stambouli O, Gruner N, Marcus U, Cai W, Zhang W, et al Detection of infections with hepatitis B virus, hepatitis C virus, and human immunodeficiency virus by analyses of dried blood spots—performance characteristics of the ARCHITECT system and two commercial assays for nucleic acid amplification. Virol J. 2013;10:72 doi: 10.1186/1743-422X-10-72 ; PubMed Central PMCID: PMCPMC3599381. 23497102 10.1186/1743-422X-10-72 PMC3599381

[21] Villar LM, de Oliveira JC, Cruz HM, Yoshida CF, Lampe E, Lewis-Ximenez LL. Assessment of dried blood spot samples as a simple method for detection of hepatitis B virus markers. J Med Virol. 2011;83(9):1522–9. doi: 10.1002/jmv.22138 . 21739441 10.1002/jmv.22138

[22] Kania D, Bekale AM, Nagot N, Mondain AM, Ottomani L, Meda N, et al Combining rapid diagnostic tests and dried blood spot assays for point-of-care testing of human immunodeficiency virus, hepatitis B and hepatitis C infections in Burkina Faso, West Africa. Clin Microbiol Infect. 2013;19(12):E533–41. doi: 10.1111/1469-0691.12292 . 23902574 10.1111/1469-0691.12292

[23] Lee CE, Sri Ponnampalavanar S, Syed Omar SF, Mahadeva S, Ong LY, Kamarulzaman A. Evaluation of the dried blood spot (DBS) collection method as a tool for detection of HIV Ag/Ab, HBsAg, anti-HBs and anti-HCV in a Malaysian tertiary referral hospital. Ann Acad Med Singapore. 2011;40(10):448–53. . 22206053

[24] Mendy M, Kirk GD, van der Sande M, Jeng-Barry A, Lesi OA, Hainaut P, et al Hepatitis B surface antigenaemia and alpha-foetoprotein detection from dried blood spots: applications to field-based studies and to clinical care in hepatitis B virus endemic areas. J Viral Hepat. 2005;12(6):642–7. doi: 10.1111/j.1365-2893.2005.00641.x . 16255766 10.1111/j.1365-2893.2005.00641.x

[25] Wayal S, Llewellyn C, Smith H, Fisher M. Home sampling kits for sexually transmitted infections: preferences and concerns of men who have sex with men. Cult Health Sex. 2011;13(3):343–53. doi: 10.1080/13691058.2010.535018 . 21154069 10.1080/13691058.2010.535018

[26] Smit PW, van der Vlis T, Mabey D, Changalucha J, Mngara J, Clark BD, et al The development and validation of dried blood spots for external quality assurance of syphilis serology. BMC Infect Dis. 2013;13:102 doi: 10.1186/1471-2334-13-102 ; PubMed Central PMCID: PMCPMC3586363. 23442198 10.1186/1471-2334-13-102 PMC3586363

[27] Thompson AJ, Nguyen T, Iser D, Ayres A, Jackson K, Littlejohn M, et al Serum hepatitis B surface antigen and hepatitis B e antigen titers: disease phase influences correlation with viral load and intrahepatic hepatitis B virus markers. Hepatology. 2010;51(6):1933–44. doi: 10.1002/hep.23571 . 20512987 10.1002/hep.23571

[28] Tuaillon E, Lozano C, Kuster N, Poinso A, Kania D, Nagot N, et al Long-term hepatitis B virus surface antigen decay in HIV-1/hepatitis B virus-coinfected adults initiating a tenofovir-containing regimen. J Clin Microbiol. 2012;50(9):3096–8. doi: 10.1128/JCM.00971-12 ; PubMed Central PMCID: PMCPMC3421810. 22760046 10.1128/JCM.00971-12 PMC3421810

[29] Tuaillon E, Mondain AM, Nagot N, Ottomani L, Kania D, Nogue E, et al Comparison of serum HBsAg quantitation by four immunoassays, and relationships of HBsAg level with HBV replication and HBV genotypes. PLoS One. 2012;7(3):e32143 doi: 10.1371/journal.pone.0032143 ; PubMed Central PMCID: PMCPMC3293872. 22403628 10.1371/journal.pone.0032143 PMC3293872

[30] Spielberg F, Critchlow C, Vittinghoff E, Coletti AS, Sheppard H, Mayer KH, et al Home collection for frequent HIV testing: acceptability of oral fluids, dried blood spots and telephone results. HIV Early Detection Study Group. AIDS. 2000;14(12):1819–28. . 10985320 10.1097/00002030-200008180-00018

